# Evaluating CT-based de novo lumbar scoliosis model for pedicle screw placement: A simulation for enhancing surgical skills and orthopedic training

**DOI:** 10.1097/MD.0000000000047245

**Published:** 2026-01-23

**Authors:** Volga Ozturk, Anil Murat Ozturk, Figen Govsa, Mehmet Asim Ozer

**Affiliations:** a Department of Orthopaedics and Traumatology, Faculty of Medicine, Ege University, Izmir, Turkey; b Department of Anatomy, Digital Imaging and 3D Modelling Laboratory, Faculty of Medicine, Ege University, Izmir, Turkey.

**Keywords:** learning model, pedicle screw fixation, resident training, scoliosis, screw malposition, spinal fusion surgery

## Abstract

Managing adult degenerative/de novo scoliosis patients presents challenges due to the unique characteristics of each deformity, the complexity of classification. The rotation or translation of vertebrae can lead to the displacement of surrounding vital structures, narrowing the safe zones for pedicle screw insertion. This study evaluates the effectiveness of 3-dimensional (3D) scoliotic models in orthopedic training, specifically for teaching pedicle screw placement in scoliosis surgery. The goal is to validate the efficacy of a 3D model-navigated training system. Twenty life-sized 3D models of de novo scoliotic lumbar deformity were utilized by 20 residency trainees, categorized based on their years of experience: those with <3 years and those with 3 years or more. Each trainee practiced pedicle screw placement across 5 lumbar levels. Their performance was assessed through postinstrumentation CT scans for accuracy and detection of screw malpositions. Instances of screw malposition and breaches of the pedicle’s medial and lateral walls were recorded. The distribution of total cortical and critical penetrations was analyzed based on years of experience and lumbar levels. The training significantly enhanced the speed and accuracy of pedicle screw placement, reducing the incidence of errors. Trainees with less than 3 years of experience were 6 times more likely to misplace screws, particularly at higher levels of lumbar deformity. The average time to complete the simulation was approximately 34 minutes, with a 77.5% accuracy rate for screw placement. Odds ratios indicated that less experience increased the risk of critical cortex penetration 6-fold, and screw placement at the level of deformity increased the risk of poor placement by 4.7 times. The use of 3D models in surgical training enables residents to safely gain practical experience, highlighting potential for broader application in complex orthopedic procedures. This training system supports increasing task complexity and enhancing performance in actual surgical settings.

## 1. Introduction

Adult degenerative/de novo lumbar scoliosis (ADS) represents a complex 3-dimensional spinal deformity characterized by lateral curvature and vertebral axial rotation.^[1]^ Treatment focuses on halting the progression of the curvature and achieving spinal correction, which is necessary in approximately 0.2% to 0.5% of AIS cases.^[2,3]^ The gold standard for surgical intervention includes posterior spinal instrumentation and fusion utilizing pedicle screws, executed with the patient in a prone position. This approach facilitates spinal fusion by enabling the precise insertion of screws and rods following correction.^[4]^ Surgery is recommended for patients who exhibit neurological symptoms, are unresponsive to conservative treatments, or continue to experience progression of deformity despite non-surgical management efforts.^[5]^

A systematic review identified a pedicle screw breach rate of 4.2%, which escalates to 15.7% with routine postoperative CT scanning. Another review noted that complications related to screws resulted in an implant complication rate of 1.1% and a neurological deficit rate of 0.8%.^[6]^ The risk of harming vascular structures, nerve roots, and the spinal cord due to incorrect screw placement highlights the significant challenges faced in ADS surgical treatments.

Orthopedic residents are tasked with enhancing comprehensive patient care while refining their surgical and clinical skills.^[7]^ The procedure of pedicle screw placement, known for its complexity and high technical demands, presents a formidable learning curve.^[8]^ The limited visibility of spinal landmarks during surgery necessitates a profound understanding of spinal anatomy, especially of structures obscured during the operation. Even for seasoned spine surgeons, the free-hand placement of screws involves a high risk of misplacement, with error rates ranging between 20% and 43%.^[9,10]^ Additionally, incorrectly placed screws can compromise spinal stabilization, lead to construct failure, or result in pseudoarthrosis. The risks of misplacement include vertebral wall perforation, which may cause dysesthesia, neurological injury, or hemorrhage. The incidence of pedicle screw mispositioning varies from 0 to 25%, influenced by the complexity of the case and the surgeon’s experience.

In the advanced stages of their training, residents develop critical psychomotor skills necessary for high-specialty surgeries, typically performed by consultants or fellows who have specialized in a particular area.^[11]^ Simulation systems provide a stress-free learning environment that complements operating room training. These systems are designed to enhance performance through structured skill development and complexity escalation.^[12,13]^

Limited visibility of spinal landmarks during surgery necessitates a profound understanding of spinal anatomy, especially of structures that are not visible during the procedure. Even for experienced spine surgeons, free-hand screw placement carries a significant risk of incorrect placement, with reported rates ranging from 20% to 43%.^[14,15]^ The risks associated with misplacement include vertebral wall perforation, which can lead to dysesthesia, neurological injury, or hemorrhage. Additionally, misplaced screws can undermine spinal stabilization, potentially resulting in construct failure or pseudoarthrosis. The rates of pedicle screw mispositioning vary from 0% to 25%, depending on the complexity of the case and the surgeon’s level of experience.

Recent advancements in surgical training include the introduction of 3D-printed simulators, crucial for mastering safe and accurate pedicle screw instrumentation.^[16]^ This study evaluates the effectiveness of using a life-size 3D-printed scoliotic spine model in training for free-hand pedicle screw placement, focusing on improving accuracy and procedure efficiency. The assessment included surgery duration, total and critical penetration counts for each surgeon, comparing cortical and critical penetrations across years of experience, and evaluating critical cortical penetration distribution across lumbar levels in relation to neurological damage.

## 2. Material and method

### 2.1. Sample resident group

This study involved a cohort of 20 residency trainees from the Department of Orthopedics and Traumatology, University Hospital, covering the academic years 2022 to 2023. To assess the impact of surgical experience, residents were categorized into 2 groups based on their surgical experience: those with less than 3 years of experience, who typically serve as secondary surgeons in operations, and those with 3 or more years of experience, who assume the role of primary surgeons for various orthopedic procedures under the guidance of faculty members. A comparative analysis of performance metrics between these 2 groups constituted a primary objective of the study. The study was reviewed and approved by the Ethical Committee of the Ege University, Izmir (Approval No: 22-4.1T/48). Requirements for written consent was waived by the Ethical Committee. However, patients’ informed consent was obtained prior to data acquisition and patient data was pseudoanonymized. This study was approved by the Ege University. All patients signed written informed consent to participate.

### 2.2. Application

**Figure 1.**
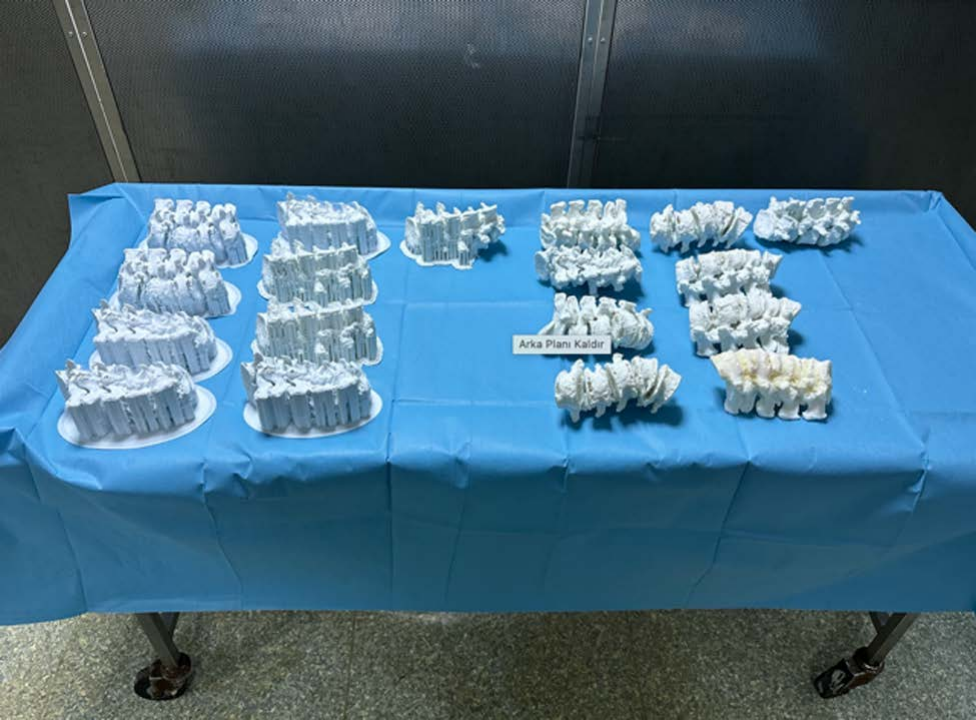
Preoperative simulator models of scoliosis with intact and removed support structures.

**Figure 2.**
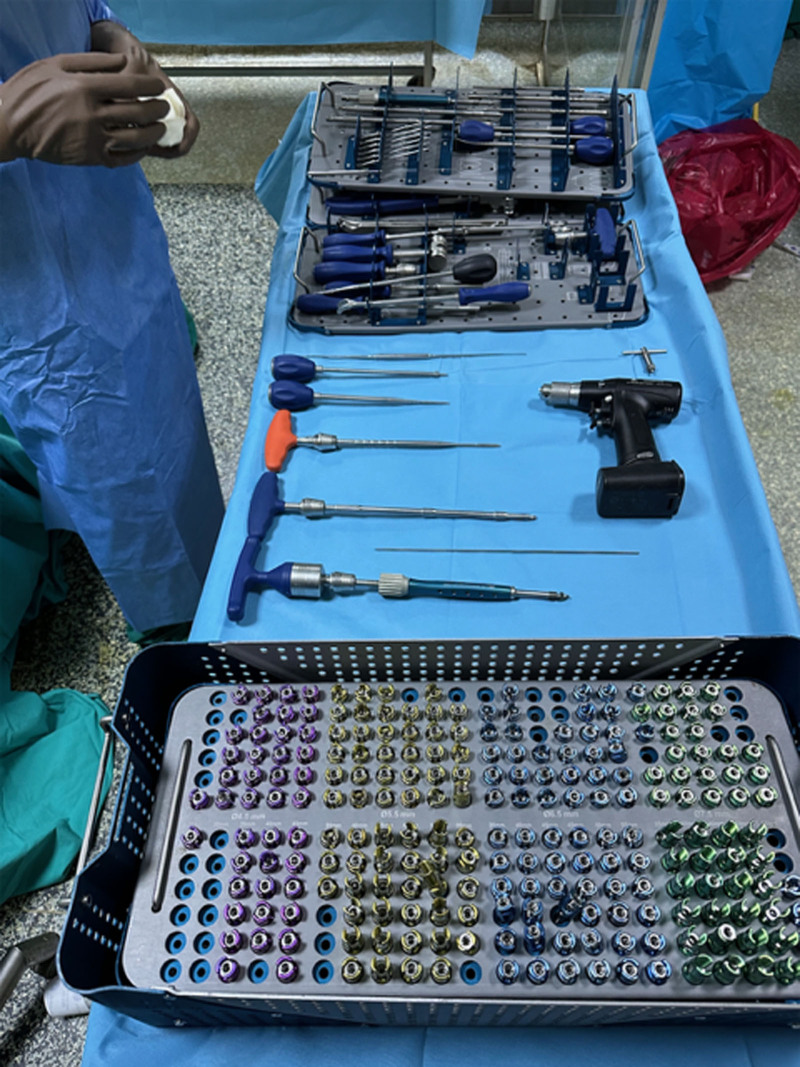
Display of pedicle screws with various lengths but the same diameter presented during the lecture.

**Figure 3.**
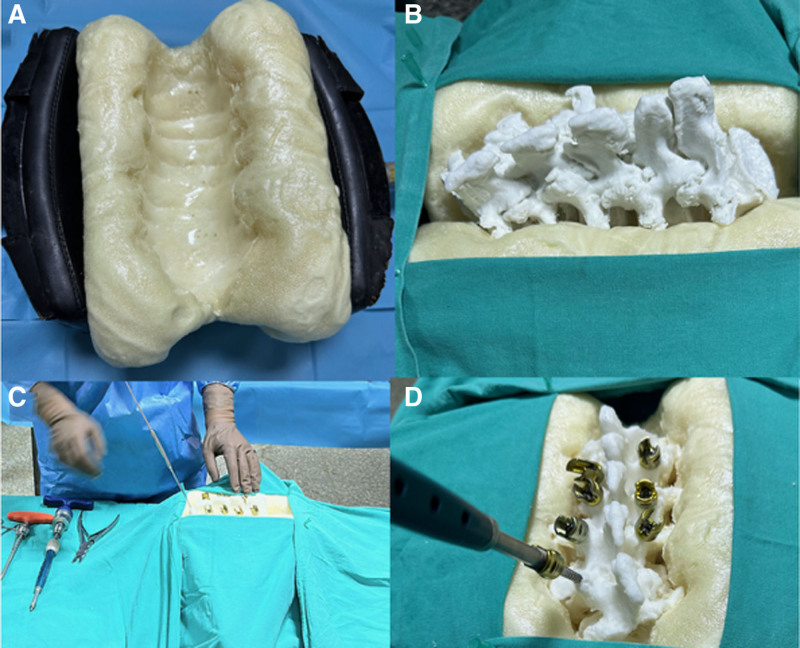
(A–D) The creation of the foundational material (A), forming the primary base for the scoliotic model (B). Preoperative planning for pedicle screw placement involves steps like identifying key orientation points and utilizing navigational tools. This planning is verified in the actual procedure (C and D) using a scoliotic model for accuracy.

Before the hands-on session, all participants attended a detailed instructional presentation delivered by an experienced spinal surgeon. This presentation covered key surgical principles, potential complications, and specific techniques for pedicle screw insertion, including the use of screws with the same diameter but different lengths (Figs. 1 and 2). Following this, each resident was tasked with inserting 10 pedicle screws across 5 lumbar levels on a life-size 3D-printed model of de novo lumbar scoliosis (Fig. 3).

### 2.3. Evaluation

The evaluation of this study concentrated on the surgical appropriateness of the procedures performed using the 3D-printed scoliotic spine model and explored their correlation with the residents’ level of surgical experience. This aimed to provide insights into the optimal timing for practical application in scoliosis surgery. Key performance metrics included the duration of each procedure, the total and critical penetration counts by each surgeon (Fig. 1).

**Figure 4.**
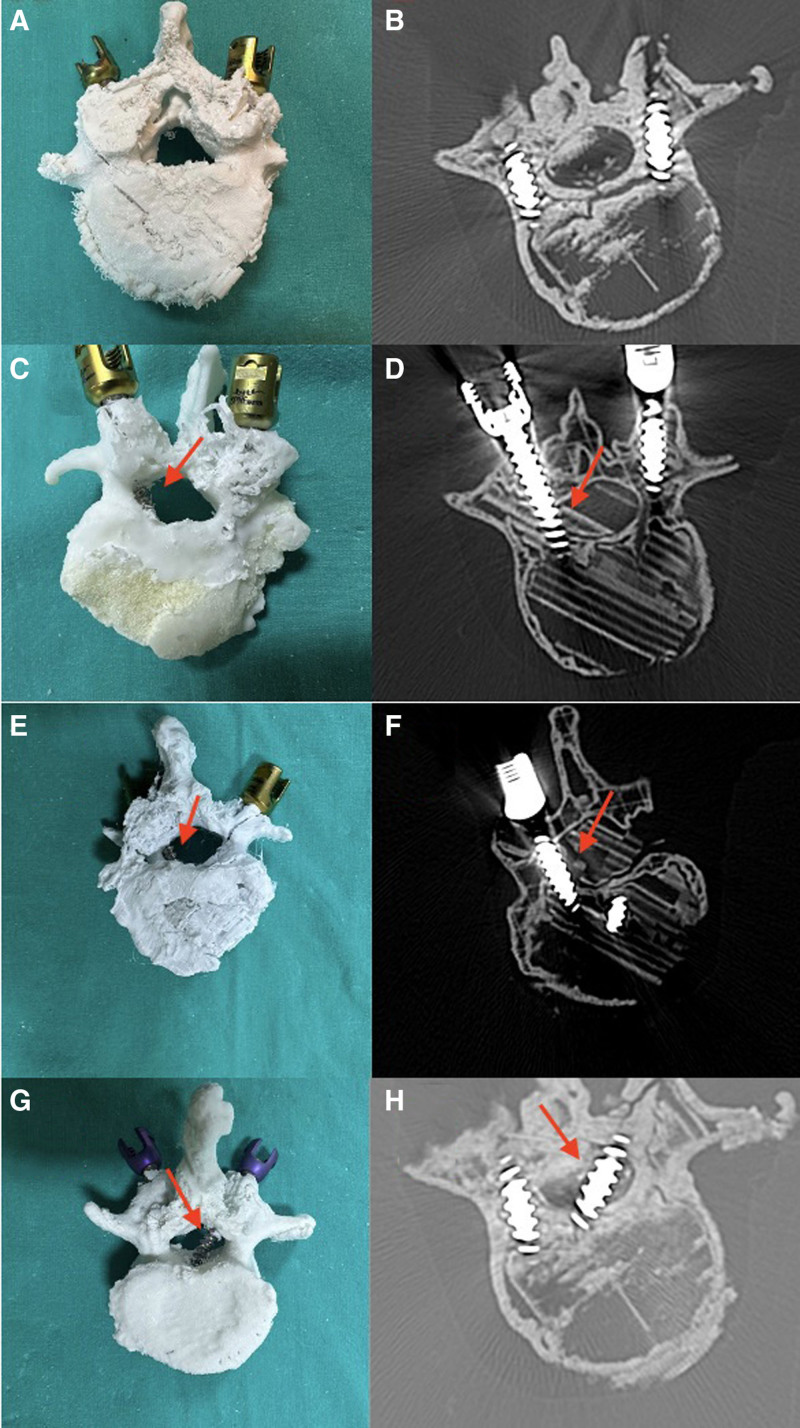
(A–H) Postoperative verification of the accurate placement of pedicle screws in a scoliotic model showing lateral curvature and vertebral axial rotation. The model (A) and CT images (B) display the insertion. Levels of penetration (arrow) are illustrated at Level 1 in (C and D) Level 2 in (E and F) and Level 3 in (G and H) Postoperative assessment of the scoliotic model in (A, C, E, G), and confirmation with CT imaging in (B, D, F, H).

**Figure 5.**
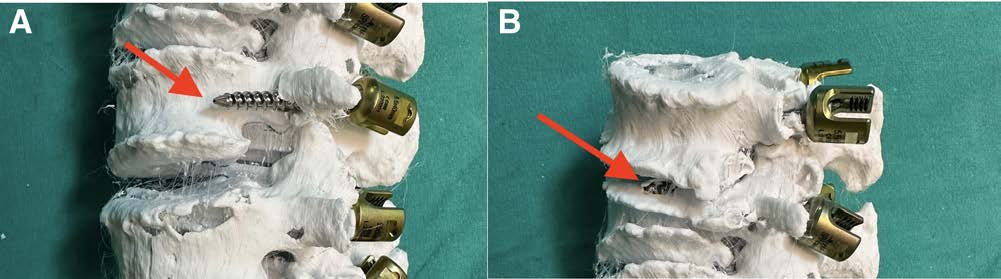
(A and B): Postoperative assessment of a scoliotic model showing lateral curvature and vertebral axial rotation with lateral penetration (arrow) shown in (A) and superior penetration (long arrow) in (B).

Penetrations were meticulously analyzed based on the lumbar vertebral levels and sides of insertion. Differences in the total number of cortical and critical penetrations (categorized as Level 2 and Level 3 screws) were statistically compared across varying years of experience, offering a longitudinal perspective on skill progression (Tables 1 and 2). Additionally, the study meticulously assessed the distribution of critical cortical penetrations, particularly those associated with neurological damage, across different lumbar levels (Table 2, Figs. 4A–H, 5A and B).

**Table 1:** Total number of penetrations by assistant’s years of experience.

Years of experience		Cortex penetration		Total	Statistical significance	Odds ratio
		Present	Absent			
≤3 Years	Screw Count (Frequency)	33 (**73.3%**)	77 (49.7%)	110 (55%)	**0.008** **	**2.786** *
>3 Years	Screw Count (Frequency)	12 (**26.7%**)	78 (50.3%)	90 (45%)		

		Level Critical Penetration		Total		
		Level II-III	Level 0-I			
≤3 yr	Screw Count (Frequency)	24 (**85.7%**)	86 (50%)	110 (55%)	**0.001** ***	**6.000** ****
>3 yr	Screw Count (Frequency)	4 (**14.3%**)	86 (50%)	90 (45%)		

* Odds ratio for years of experience: 2.786.

** Significant difference at the 0.008 level according to the Chi-Square test.

*** Significant difference at the 0.001 level according to the Chi-Square test.

**** Odds Ratio of 6.000 in terms of Years of Experience.

**Table 2:** Comparison of the malpositioned pedicle screw at the apex of deformity with other levels.

Vertebral levels	Critical penetrations screw count (frequency)		Total	Statistical significance	Odds ratio
	Present	Absent			
L2-L3	20 (**71.4%**)	60 (34.9%)	80 (40.0%)	**0.001** **	**4.667** *
L1-L4-L5	8 (**28.6%**)	112 (65.1%)	200 (100.0%)		
Total	28 (100%)	172 (100%)	200 (100.0%)		

* Odds ratio compared to other vertebral levels: 4.667.

** Significant difference at the 0.001 level according to the Chi-Square test.

### 2.4. Radiologic analysis

CT scans were performed immediately post-screw insertion to assess the accuracy of pedicle screw placement (Fig. 4B, D, F, and H). Screw positions were meticulously analyzed and categorized using the Frank screw malposition classification, as defined by Learch and Wiesner. The classification delineated screw placement into 4 categories based on their proximity to the pedicle walls:

Encroachment: screw fully contained within the pedicle.

Level I: Breach <2 mm.

Level II: Breach between 2 and 4 mm.

Level III: Breach >4 mm.

A critical violation was identified for breaches >2 mm, as perforations exceeding this threshold significantly elevate the risk of neurological complications.

### 2.5. Statistical analysis

Descriptive statistics and the Friedman test were utilized for comparative analysis of the screw placement data. Primary comparative analyses were conducted between the 2 resident groups stratified by years of experience (≤3 years vs >3 years). Following the procedures, residents received feedback based on Spearman correlation analysis, which evaluated the precision of the pedicle screw placements. Statistical significance was established at *P*-values <.05, analyzed using IBM SPSS Statistics, Version 24 (IBM Corp., Armonk).

## 3. Results

### 3.1. Scoliotic model printing

DICOM data from actual lumbar scoliosis patients’ scans were segmented and converted into a printable format using software programs, including 3D Slicer and Autodesk Meshmixer. Subsequently, the data were prepared for 3D printing with Z Suit software. The models were printed in solid form using Zortrax M200 Dual and Formlabs Form 2. The average cost to print 1 model was determined to be $12, with 316 grams of material used per model, and the printing time for a single model was 2 days, 2 hours, and 12 minutes (Figs. 1 and 3).

A foam material, created from a mixture of polyol and isocyanate, was chosen as the support material for the models due to its ease of availability and cost-effectiveness (Fig. 3A, C, and D). This foam, resulting from a one-to-one mixture of polyol and isocyanate, ensured the stability of the printed vertebral model during surgical procedures and obscured the anterior structures from the practitioner’s view (Fig. 3B). The combination of the solid, rigid PLA vertebrae and the surrounding foam simulates a rudimentary corticocancellous bone interface and provides resistance during probing and screw insertion, offering a more realistic haptic feedback compared to a bare bone model, though it does not fully replicate the compliance of live soft tissues.

### 3.2. Surgical method

All 20 residents participating in the study, who were all male, proceeded to simulate real patient scenarios in the operating room with the scoliotic models prepared in the prone position. Each participant was required to perform pedicle screw placement on 10 predetermined locations (Figs. 3–5).

*The simulation of the surgery on the protocol included the following steps:*

*First, the rod length was determined on model.

*Identification of the longitudinal surgical access point of the spinous processes.

*Following anatomical landmarks such as facet joints, transverse processes, and the lateral portion of the pars interarticularis were demonstrated.

*After determining the entry point at the junction where the major axis of the transverse process meets the line through the lateral margin of the superior facet joint, a cortical breach was created and a bone probe placed (Fig. 3C).

*The bone probe was navigated through the pedicle along the ideal trajectory, aiming towards the contralateral transverse process to align the screw with the superior endplate. A probe was then inserted to check the integrity of the hole walls.

*With a guidewire, the optimal direction angle for the pedicle screw implantation was decided. Subsequently, drilling with a small-diameter drill into the vertebrae was performed to make a hole and the drilling was continued with tappers up to 5mm.

*The chosen screw was inserted, ensuring it passed through the pedicle canal and penetrated 2/3 of the vertebral body’s depth (Fig. 4A–H).

*Classification of pedicle screw placement according to the degree of possible pedicle wall violation under CT control (Fig 4B, D, F, and H).

### 3.3. Evaluation of residents’ pedicle screw placements

The average duration required to complete the pedicle screw placement was approximately 34 minutes and 36 seconds. Completion times varied, with the quickest being 26 minutes and 22 seconds and the longest being 62 minutes and 59 seconds, reflecting the diverse skill levels among the participants. The residents with <3 years of experience completed the procedure in an average of 26 minutes and 42 seconds, while those with more than 3 years of experience took 36 minutes and 37 seconds. We believe the longer completion time for the more experienced residents is due to the fact that 2 surgeons in this group were unsatisfied with the placement of a total of 8 screws and decided to revise them, which extended the procedure time. No statistically significant difference was found between the procedure completion time, years of surgical experience, and the amount of medial wall penetration. Out of 200 screws placed, 77.5% were positioned correctly. However, there were 45 instances of incorrect placements, with the most frequent error being the penetration of the medial cortex, which poses risks of neurological complications (Fig. 2C–H). Notably, the most precise resident achieved a flawless performance with no errors, while another struggled with 5 critical errors, 4 of which were classified as severe due to the high risk of causing neurological damage.

#### 3.3.1. Evaluation of residents’ pedicle screw placements

The evaluation of pedicle screw placement revealed that 17 screws (8.5%) were classified as Level 1 penetrations, displaying breaches of <2 mm (Fig. 4C and D), 3 screws (1.5%) as Level 2 (Fig. 4E and F), and 25 screws (12.5%) as Level 3, indicating breaches >4mm (Figs. 3 and 4G,H). Analysis of cortical penetration by years of residency showed that residents with <3 years of experience were responsible for 33 cortical penetrations, compared to only 12 by more experienced residents. This marked difference (*P* = .008) suggested a 2.786-fold increase in the risk of cortical penetration among less experienced residents. Moreover, the likelihood of critical cortical penetration (Level 2 and Level 3) was notably higher (*P* = .001) in this group, presenting a 6-fold increased risk. The orientation of cortical penetrations varied significantly between the 2 groups, with medial penetrations observed exclusively in residents with over 3 years of experience (Table 1).

The distribution of critical cortical penetrations across various lumbar levels was closely scrutinized. Of the misaligned screws, 20 were located at the apex of the deformity, primarily at the L2 and L3 levels, with the remaining 8 found at the L1, L4, and L5 levels (Table 2). A significant disparity (*P* = .001) was observed in the incidence of critical cortical perforations across these levels, suggesting a 4.667 times greater risk of misplacement at the deformation levels. Remarkably, only one of the 28 improperly placed screws was at the L5 level, which exhibited no deformity and maintained normal alignment. The vast majority, 27 screws, were distributed across the L1, L2, L3, and L4 levels, with statistical significance noted (*P* = .037). Additionally, an Odds ratio calculation indicated that placing a screw in a vertebra with no deformity reduced the risk of poor placement by 7.93 times.

## 4. Discussion

The practice of inserting pedicle screws, particularly in scoliosis patients, presents inherent risks, primarily due to the potential for spinal cord injury when engaging with the pedicles of rotated lumbar vertebrae (Figs. 4A–H, 5A and B).^[4,6]^ Complications are further exacerbated by factors such as the asymmetrical nature of pedicles and the deviation of the spinal cord within the spinal canal in scoliotic spines, which challenge the safe and accurate placement of screws. The feasibility of screw insertion is contingent not only on the pedicle channel’s width but also on the degree of spinal deviation, necessitating meticulous preoperative planning and execution (Figs. 4A–H, 5A and B).

Given the technical demands and the need for deep anatomical knowledge, training for precise pedicle screw placement is crucial.^[17]^ Traditional cadaveric training, while beneficial, faces logistical challenges including limited availability of suitable cadavers, especially those with specific pathologies or deformities that mirror the clinical situations trainees may encounter.^[18]^ Although studies indicate that training with cadavers and sawbones can diminish the rate of suboptimal screw placements, the scarcity of cadavers hampers the scalability of such training methods.

The integration of advanced orthopedic simulation programs into surgical training has marked a significant progression, enhancing the precision of screw placements. Moreover, emerging technologies and accessible tools, such as software applications, animations, and mobile applications, are being leveraged to augment the training of surgical residents.^[19,20]^ The advent of computer navigation-assisted surgery, coupled with developments in virtual reality and haptic feedback simulations, provides residents with unprecedented interactive learning opportunities.^[21]^ These technologies facilitate a deeper understanding of complex surgical dynamics, such as cement leakage during vertebroplasty.^[22]^ Furthermore, the utilization of advanced imaging techniques like 2- and 3-dimensional fluoroscopy and intraoperative CT enables real-time navigation tailored to the patient’s specific anatomy, significantly outperforming conventional free-hand methods in terms of accuracy and safety.^[22–25]^

**Figure 6.**
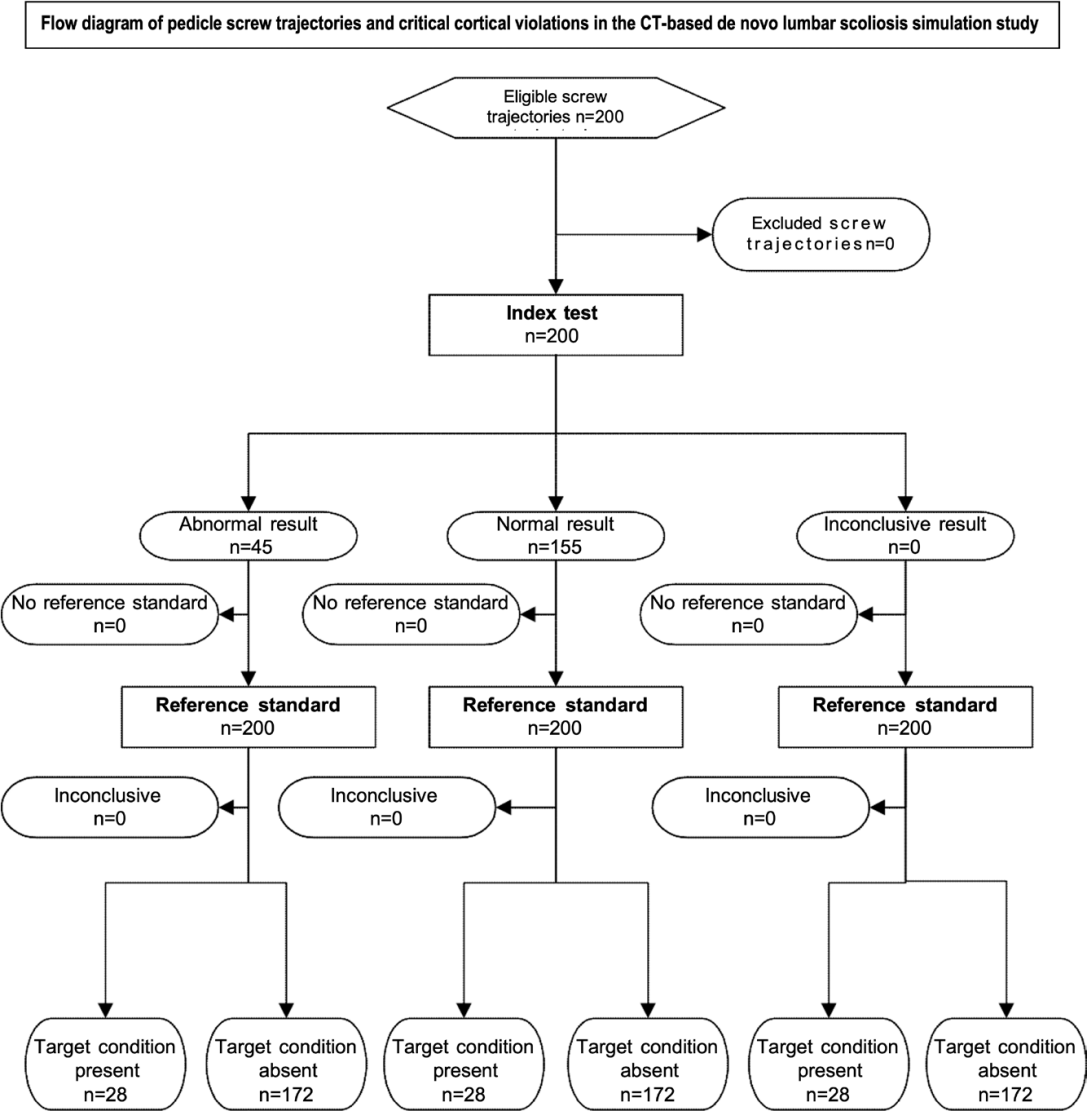
Flow diagrams of this study.

This study introduces innovative approaches to orthopedic and spine surgery training, particularly emphasizing the utility of 3D models for assessing performance in pedicle screw placement (Fig. 6). It underscores the potential of well-designed, cost-effective training modules to enhance technical skills and proficiency in simulated procedures for lumbar pedicle screw placement. Utilizing low-cost 3D printing and software technologies, the study created affordable, life-sized spinal models with scoliotic deformities. These models could be produced quickly and on a large scale, facilitating their use in operating room settings for spinal surgeons to hone their skills (Figs. 1-5).

The scoliotic spine models, with their complex anatomical structure and inherent rotation, presented a high risk of screw penetration (22.5%) as demonstrated in this study (Tables 1 and 2). The research also explored the impact of manual dexterity and the accumulated years of experience as a surgical assistant on the likelihood of successful outcomes in scoliosis surgery (Table 1). When comparing orthopedic surgeons with less than 3 years of surgical training to those with 3 years or more, significant differences emerged in the rates of cortical and critical penetration during the fixation of scoliotic lumbar vertebrae, with *P*-values of .008 and .001, respectively (Fig. 6, Table 1). It was found that surgeons with less than 3 years of experience had a 2.8 times higher risk of cortical penetration and a 6-fold higher risk of critical perforations potentially leading to neurological damage (Table 1). Penetration towards the medial direction was most commonly observed (16%), highlighting a specific area for focused improvement in training protocols (Table 1).

Given that none of the participating residents had previously performed spinal surgery as the lead surgeon, practice with these 3D models could significantly reduce the learning curve associated with vertebral surgery. The analysis further revealed that the risk of screw penetration in vertebrae with scoliosis deformities was increased by 4.7 times, whereas interventions on a non-deformed spine in the scoliotic model were found to be 7.9 times safer (Fig. 6, Table 2).

Training on scoliotic models has proven valuable for educational purposes, demonstrating a marked increase in the accuracy of screw placements and a decrease in pedicle infractions with repeated practice. However, the realism and full replication of the surgical field are areas that require further enhancement. Simulation training is increasingly recognized for its role in providing trainees with the opportunity to develop foundational skills within a risk-free environment, ultimately preparing them for complex, high-stakes scenarios encountered in the real world (Table 1).

Pedicle screw fixation has become the gold standard for spinal fusion procedures, yet it is not without risks. Complications such as nerve damage, failure of the spinal construct, and deep wound infections have been documented. Additionally, malposition, particularly in deformity corrections, can result in severe complications, such as major vascular injury, which may be life-threatening.^[26,27]^ Therefore, ensuring that pedicle screw placement is performed using safe and precise techniques is critical to minimizing these risks.^[28]^

Experience is a critical factor in enhancing surgical outcomes across various medical procedures, with a well-documented correlation between increased case volumes and improved clinical results. This relationship is particularly evident in pedicle screw placement, where a surgeon’s accumulated experience is inversely related to the rate of complications. It is advisable for novice spine surgeons to perform their initial placements of pedicle screws, particularly in less complicated cases, under the supervision of seasoned practitioners to minimize risks and errors.

### 4.1. Strength point

The university hospital where the research is conducted is a highly respected institution in our country, known for its extensive research activities and substantial patient capacity. Our faculty has access to a Digital Imaging and 3-Dimensional Modelling Laboratory with 3D printers in the Department of Anatomy, and one of the authors (MAO) is experienced and knowledgeable in both software and hardware aspects of printing the desired model. The average cost to print 1 model was determined to be $12, with 316 grams of material used per model, and the printing time for a single model was 2 days, 2 hours, and 12 minutes (Figs. 1, 3 and 6). Consequently, we can provide this type of training to resident physicians at minimal cost, covering only the expenses for PLA raw materials. After achieving a certain level of error-free practice, residents are allowed to proceed to real surgical applications under the supervision of an instructor. Having such workshops in large hospitals paves the way for them to become attraction centers, both due to patient satisfaction and the institution’s success.

### 4.2. Limitations

The present study has several limitations. First, the study included 20 residents, and this limited sample size may restrict the generalizability of the findings. In the future, multicenter studies with larger sample sizes could increase the strength of the results. Second, collaboration across different spinal surgery centers could provide a broader range of insights and practices. Third, the study data highlighted the heightened risk of spinal cord injury and discrepancies between the spinal cord’s positioning during surgery and its depiction in post-myelography CT images. To consolidate these preliminary findings and develop robust training protocols, further research with larger participant cohorts and extended follow-up assessments of real patients is crucial.

Building on these initial findings, a critical next step will be to conduct a comparative study. A prospective trial where 1 cohort of trainees uses this patient-specific 3D scoliosis model and another uses traditional sawbones models would directly measure the relative improvement in surgical accuracy, confidence, and the reduction of the learning curve. This would provide robust evidence for the specific advantages of pathological model-based training over standard methods.

The study included 20 residents, and this limited sample size may restrict the generalizability of the findings. In the future, multicenter studies with larger sample sizes could increase the strength of the results. This study evaluated the short-term effects of simulation training, but the long-term impact on the residents’ actual surgical performance was not followed. Future studies should aim to track the long-term effects on real surgical applications. The study only assessed pedicle screw placement accuracy and operation times. Future research could evaluate the impact of training on various other surgical skills as well.

Moreover, to the best of our knowledge, this is the first study to evaluate the impact of 3D de novo lumbar scoliotic models on the surgical skills of residents at various stages of their training (Fig 6). We have not encountered a study comparing 3D training with traditional training methods in the application of CT-based de novo lumbar scoliosis models for pedicle screw placement. We hope to see publications from other researchers on this topic in the near future.

## 5. Conclusion

The integration of 3D technology-based models as teaching tools has become increasingly significant in postgraduate medical education and continuous professional development. These 3D models, particularly proficient in replicating bone rotational deformities, provide surgeons with valuable opportunities to simulate and rehearse diverse clinical scenarios prior to performing actual surgeries. In the near future, the creation and endorsement of alternative educational methods, such as 3D models, will become essential. This pilot study highlighted the effectiveness of using a scoliosis model-assisted orthopedic training system for junior trainees, showcasing its ability to improve both the precision and efficiency of surgical instruction. Nevertheless, we maintain that simulation-based training can establish a crucial foundation for surgeons as they transition into real-life patient care, allowing them to hone practical skills by rehearsing possible scenarios and complications before engaging in actual surgical procedures. We stress that such training approaches could be particularly advantageous during the shift to patient-centered clinical applications.

## Author contributions

**Conceptualization:** Volga Ozturk.

**Data curation:** Volga Ozturk, Anil Murat Ozturk, Figen Govsa, Mehmet Asim Ozer.

**Formal analysis:** Volga Ozturk, Anil Murat Ozturk, Figen Govsa, Mehmet Asim Ozer.

**Investigation:** Volga Ozturk, Anil Murat Ozturk.

**Methodology:** Volga Ozturk, Anil Murat Ozturk, Figen Govsa, Mehmet Asim Ozer.

**Project administration:** Mehmet Asim Ozer.

**Resources:** Figen Govsa.

**Supervision:** Anil Murat Ozturk, Figen Govsa, Mehmet Asim Ozer.

**Validation:** Anil Murat Ozturk.

**Visualization:** Volga Ozturk, Anil Murat Ozturk, Figen Govsa, Mehmet Asim Ozer.

**Writing – review & editing:** Figen Govsa, Mehmet Asim Ozer.

**Writing – original draft:** Anil Murat Ozturk, Figen Govsa, Mehmet Asim Ozer.
